# Gabapentin for uremic pruritus in hemodialysis patients: a qualitative systematic review

**DOI:** 10.1186/s40697-016-0107-8

**Published:** 2016-03-28

**Authors:** Torey Lau, Sharon Leung, Wynnie Lau

**Affiliations:** Vancouver General Hospital, Lower Mainland Pharmacy Services, 899 West 12th Avenue, Vancouver, BC V5Z 1M9 Canada; St. Paul’s Hospital, Lower Mainland Pharmacy Services, 1081 Burrard St., Vancouver, BC V6Z 1Y6 Canada

**Keywords:** Hemodialysis, Uremic pruritus, Gabapentin, Pruritus, Itch

## Abstract

**Purpose of the review:**

Uremic pruritus (UP) is a common discomfort of dialysis-dependent end-stage renal disease. Some studies suggest a neuropathic cause of UP. Gabapentin, an anticonvulsant, has shown promising results as an emerging drug to treat this condition.

**Objective:**

An updated qualitative systematic review was conducted to evaluate its efficacy and safety in hemodialysis patients.

**Source of information:**

Ovid MEDLINE, EMBASE, Cochrane Central Register of Controlled Trials, Clinicaltrials.gov, and Google Scholar through June 2015 were used as sources of information.

**Patients:**

Patients are adult hemodialysis patients receiving gabapentin for UP.

**Methods:**

All randomized controlled trials (RCTs), quasi-RCTs, observational studies, open-label studies, and retrospective studies were included. Case series and case reports were excluded. All descriptions and data were extracted independently by two authors.

**Results:**

Seven studies evaluating gabapentin with a total of 179 patients were included. Most patients were refractory to antihistamines and topical emollients. Statistically significant favorable outcomes on pruritus scores were found in six studies. Five studies evaluated antipruritic efficacy based on a 10-point visual analog scale (VAS), and improvements in the range of an absolute decrease of 5.7 to 9.4 points from baseline were achieved on average by 3–8 weeks of treatment. Side effects are common with six studies reporting at least 26 incidences of side effects such as somnolence, dizziness, and fatigue. A total of four patients reportedly discontinued gabapentin due to intolerability.

**Limitations:**

Our review is limited by the inclusion of generally small, lower quality studies that lacked comparator groups or were open-label studies. Since the first two randomized controlled trials were published, no further high-quality studies have been conducted.

**Implications:**

Our review supports a trial of gabapentin for the management of UP in hemodialysis patients refractory to antihistamines and/or emollients. The results should be interpreted cautiously due to the lower quality of included studies. We recommend a starting dose of 100 mg orally after hemodialysis to minimize adverse events in this population.

## Abrégé

**Mise en contexte:**

Le prurit urémique est un symptôme clinique fréquemment associé à l’insuffisance rénale chronique et des études suggèrent qu’il serait d’origine neuropathique. Un anticonvulsivant, la gabapentine, a démontré des résultats prometteurs et pourrait s’avérer la pharmacothérapie d’avenir pour aider à soulager ce symptôme.

**Objectif de la revue:**

Cette revue visait à démontrer l’innocuité de la gabapentine et à évaluer son efficacité dans le traitement du prurit urémique chez les patients dialysés.

**Participants:**

Il s’agit de patients dialysés adultes recevant de la gabapentine pour le soulagement du prurit urémique associé à leur état.

**Méthode et sources:**

Cette étude systématique a été réalisée par le passage en revue des bases de données sur Ovid MEDLINE et EMBASE ainsi que par la consultation du *Cochrane Central Register of Controlled Trials*, du site internet *Clinitrials.gov* et de *Google Scholar* jusqu’en juin 2015. La revue inclut tous les essais cliniques randomisés et quasi randomisés, les études observationnelles, les études ouvertes et les études rétrospectives répertoriées dans les sources susmentionnées. Toutes les descriptions et les données utilisées dans cette revue ont été recueillies par deux des auteurs, de façon indépendante. Les rapports de cas ainsi que les séries de cas relevés dans les sources consultées n’ont pas été retenus.

**Résultats:**

La revue porte sur un total de 179 patients répartis dans sept études traitant de l’effet de la gabapentine pour le traitement du prurit urémique. La plupart des patients suivaient ce traitement à la suite de l’échec des traitements par les antihistaminiques et les émollients. Des résultats positifs et statistiquement significatifs ont été répertoriés dans six des sept études passées en revue. Une échelle visuelle analogique en 10 points a servi pour l’évaluation de l’efficacité antiprurigineuse de la gabapentine dans cinq des sept études. Une baisse absolue de la sévérité du prurit allant de 5,7 à 9,4 points a été notée par rapport aux valeurs initiales, et ce pour une période moyenne de traitement de 3 à 8 semaines. En ce qui concerne les effets secondaires fréquemment associés à un traitement à la gabapentine (somnolence, étourdissement ou asthénie), ils ont été rapportés à 26 reprises parmi six des sept études révisées. De plus, quatre patients ont dû cesser la prise de gabapentine en raison d’une intolérance au médicament.

**Limites de l’étude:**

La présente revue repose sur des données recueillies à partir de la consultation d’un nombre restreint d’études. La majorité des études incluses dans la revue n’étaient pas de grande envergure et s’avéraient peu rigoureuses au plan scientifique : certaines ne comportant pas de groupe comparateur, d’autres consistant en des études ouvertes. Parmi les sept études passées en revue, seuls deux essais cliniques randomisés avaient été publiés et aucune autre étude rigoureuse n’a été réalisée depuis.

**Conséquences:**

Cette revue recommande un essai de gabapentine pour soulager le prurit urémique chez les patients dialysés qui ne répondent pas aux traitements par les antihistaminiques et les émollients. Toutefois, l’interprétation des résultats doit être faite avec prudence en raison du manque de rigueur associé à certains types d’études incluses dans la revue. Nous suggérons une dose initiale de 100 mg de gabapentine, administrée par voie orale après la séance d’hémodialyse, afin de prévenir les effets secondaires du médicament dans cette population.

## Why is this review important?

Uremic pruritus is a common discomfort of hemodialysis patients. Gabapentin is an emerging treatment with increasing investigation of its efficacy and safety in these patients. Our qualitative analysis characterizes the current evidence available for gabapentin to manage uremic pruritus.

## Key messages

Gabapentin is likely effective for uremic pruritus but adverse events are common. Starting at a low dose of 100 mg orally after hemodialysis and titrating to effect may best provide effective and safe outcomes.

## Introduction

Uremic pruritus (UP) is a common discomfort of dialysis-dependent end-stage renal disease (ESRD). A global cross-sectional study of 18,801 hemodialysis (HD) patients found 42 % of patients experience moderate to extreme pruritus, with significant associations with decreased quality of life, poor sleep, depression, and increased mortality [1]. UP is characterized by daily itching bouts of symmetrical distribution with increasing intensity at night [2]. The pruritus may be generalized or localized, in particular, to the head, arms, back, and abdomen [2]. Despite its high incidence and significant impact on HD patients, the pathophysiology of UP is poorly understood.

Various pathophysiological mechanisms have been proposed, including skin xerosis, histamine binding to the H_4_ receptor, xenobiotic agents, and the accumulation of uremic toxins [2, 3]. Neuropathic changes in ESRD is one of the newer mechanisms explored [4]. UP is thought to be of central origin, where uremic toxins may cause neuropathy and central sensitization to itch [2]. This proposed mechanism is further supported by the correlation between itch and pain, as both are conveyed by C-fibers in the dorsal horns and transmitted to the thalamus and somatosensory cortex via the lateral spinothalamic tract [2].

Gabapentin is an antiepileptic agent that has analgesic properties in neuropathic pain [3]. Its specific pharmacological mechanism is poorly understood but its involvement in inhibiting neuronal calcium influx may interrupt the series of neuropathic events that lead to pruritic sensation in uremia [5]. It is an emerging drug for UP with an increasing number of studies published investigating its efficacy and safety for this condition. No major renal societies have created guidelines for the management of UP in hemodialysis patients, but gabapentin is recognized as a second- or third-line agent for generalized UP refractory to topical emollients and/or oral antihistamines [6]. As gabapentin is renally eliminated, its significantly increased half-life in HD patients is concerning. As several studies have been published since the most recent systematic review by Vila et al. in 2008, we sought to conduct an updated qualitative systematic review investigating the efficacy and safety of gabapentin for UP in HD patients [7].

## Methods

Studies were included in our systematic review if they were published randomized controlled trials (RCTs), quasi-RCTs, observational studies, open-label studies, or retrospective studies of any length of follow-up. Case series or case reports were excluded from our review. All studies evaluating gabapentin, with or without a comparator group for UP in adult HD patients, were included. Studies were obtained by searching in Ovid MEDLINE (1946–2015 June 19), EMBASE (1974–2015 June 19), Cochrane Central Register of Controlled Trials (June 2015), Clinicaltrials.gov (June 2015), and Google Scholar (June 2015) using the search strategy outlined in the Appendix. All publication types (e.g., conference abstracts or trial registries) and unpublished literature meeting our inclusion and exclusion criteria were considered. Reference lists of all identified articles were manually searched. We restricted our search to English-only studies.

Titles, abstracts, and full-text when needed were screened by TL and WL independently based on the above inclusion criteria. If disagreements occurred over the inclusion of studies, the two authors discussed their reasons for their decision until a consensus is reached. If a consensus could not be reached, SL independently assessed the study and made the final decision.

Studies were classified by their quality of evidence adapted from the 1996 United States Preventive Services Task Force classification system [8]. Level I studies are randomized controlled trials. Level II-1 studies are controlled trials (either as self-controls or with a comparator group) without randomization and level II-2 studies are cohort or case control studies. Level II-3 studies are multiple time series with or without intervention and level III evidences are descriptive studies, case reports, or expert opinion. TL and WL independently extracted and confirmed the data presented in this qualitative review. Any outcomes assessing efficacy or safety of gabapentin in the HD population were extracted and described. Authors of studies were contacted when data was missing or ambiguous. No funding was received for our systematic review.

Study quality was assessed using the Jadad scale for randomized controlled trials, with scores ranging from 0 to 5 [9]. For cohort studies, the Newcastle Ottawa scale was used to assess risk of bias on a scale ranging from 0 to 9 [10]. Level of agreement on study eligibility and quality assessment was tested using kappa statistic.

## Results

**Fig. 1:**
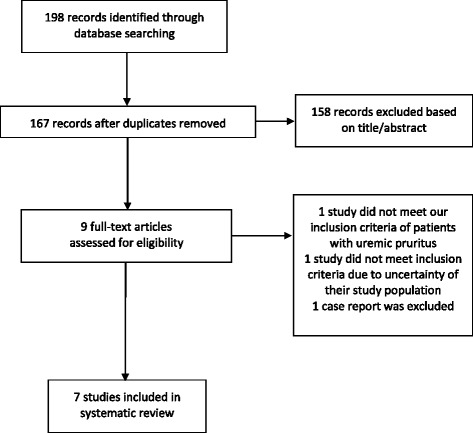
Search strategy flow diagram

Figure 1 depicts a PRISMA flow diagram of our study selection process. Of the 198 studies identified from the database search, 9 full-text articles were screened for eligibility. Upon further review, we excluded 1 case report [11], and 1 trial studying patients with neuropathy or neuropathic pain that did not meet our inclusion criteria (kappa = 1, perfect) [12]. One study published in Persian had an English abstract; but based on the abstract alone, we could not determine if the population studied were HD patients and was not included in our review [13]. We contacted the authors but did not receive a response up until the time of publication. The characteristics of the final 7 studies with a total of 179 patients included in the systematic review are depicted in Table 1. Where studies compared gabapentin to alternative agents [14, 15], or compared HD patients to non-HD patients [16], only data from the relevant arm were extracted and discussed below.

**Table 1:** Characteristics of included studies

Authors (year)	Design	Patient characteristics	Previous medication trialed	Exclusion	Gabapentin regimen	Efficacy outcomes	Quality assessment	Safety outcomes (*n*)
Level I evidence								
Gunal et al. (2004) [17]	R DB PC cross-over	*N* = 25 HD 3×/week, 4–5-h sessions Kt/V 1.37 ± 0.35 (0.5–1.93) Males 56.0 % Mean age 55 ± 11 (32–77) Baseline: Ca 2.15 ± 0.23 mmol/L PO_4_ 1.45 ± 0.39 mmol/L iPTH 20.5 ± 14.3 pmol/L	Refractory to antihistamines, nicergoline, moisturizers	NR	Gabapentin 300 mg 3× weekly post-HD × 4 weeks, then 1-week washout, then placebo × 4 weeks vs. the reverse	In chronological order: Baseline VAS (0–10) 8.5 ± 0.94 After 4 weeks placebo 7.6 ± 2.6 (*p* = 0.098) After 1-week washout 7.9 ± 1.1 After 4 weeks gabapentin 1.2 ± 1.8 (*p* = 0.0001) Follow-up: 9 weeks	3^a^	Mild to moderate somnolence, dizziness, fatigue
Naini et al. (2007) [19]	RCT DB PC	*N* = 34 HD 2×/week Males 47.1 % Mean age 62 ± 10 (43–81)	Refractory to antihistamines	Hgb < 70 g/L, PTH > 33 pmol/L, PO4 > 2.26 mmol/L, Other skin disease	Gabapentin 400 mg 2× weekly post-HD vs. placebo × 4 weeks	Baseline VAS (0–10) 7.2 ± 2.3 After 4 weeks 6.7 ± 2.6 vs. 1.5 ± 1.8 (*p* < 0.001) Follow-up: 4 weeks	4^a^	Mild to moderate somnolence, dizziness, nausea
Tol et al. (2010) [20]	R blinded cross-over	*N* = 14 HD 3×/week, 4–5-h sessions Kt/V 1.33 ± 0.17 (1.0–1.7) Males 50 % Mean age 59.7 ± 17.2 (41–88) Baseline: Ca 2.23 ± 0.18 mmol/L PO4 1.62 ± 0.29 mmol/L iPTH 31.68 ± 12.87 pmol/L	Refractory to antihistamines, nicergoline, moisturizers	<18 years old, pregnant/lactating	Gabapentin 300 mg 3× weekly post-HD × 8 weeks, then 1-week washout, then placebo (unknown duration)	Pruritus VAS score (0–10): Before gabapentin 7.6 ± 1.2 After gabapentin 1.3 ± 1.4 (*p* < 0.01) Follow-up: NR	2^a^	No side effects observed
Level II-1 evidence								
Razeghi et al. (2009) [18]	DB PC single-arm cross-over	*N* = 34 HD 3×/week, 4-h sessions Kt/V 1.31 ± 0.2 Males 23 % Mean age 58.4 ± 12.5 (28–73) Baseline: Ca 2.38 ± 0.63 mmol/L PO4 2.02 ± 0.75 mmol/L iPTH 12.2 ± 11.33 pmol/L	Refractory to antihistamines and moisturizers	Skin lesions, metabolic diseases, drug allergies, non-compliance	Gabapentin 100 mg 3× weekly post-HD × 4 weeks, then 1-week washout, then placebo × 4 weeks	In chronological order: Baseline VAS (0–100) 100 After 4 weeks gabapentin 6.4 ± 8.5 (*p* < 0.001) After 1-week washout 15 ± 11.3 (*p* < 0.001) After 4 weeks placebo 81.9 ± 11.1 (*p* < 0.001) Follow-up: 9 weeks	2^a^	Dizziness, drowsiness, and fatigue (2)
Marquez et al. (2012) [14]	Quasi-randomize, OL cross-over	*N* = 19 HD 3×/week, 4-h sessions Kt/V 1.23 ± 0.3 Males: NR Mean age 54 ± 18 Baseline: Ca 2.35 ± 0.3 mmol/L PO4 1.65 ± 0.53 mmol/L iPTH 5.79 ± 5.0 pmol/L	NR	Chronic skin or liver diseases, malignancy, chronic opiates or corticosteroids	Gabapentin 300 mg 3× weekly post-HD × 3 weeks, then 1-week washout, then desloratadine 5 mg 3× weekly × 3 weeks, vs. the reverse	Baseline VAS (0–10) 5.95 After 3 weeks gabapentin 4.6 (*p* = 0.07) After 3 weeks desloratadine 3.44 (*p* = 0.004) Follow-up: 7 weeks	1^a^	Fatigue and somnolence (9) Medication discontinuation (4)
Level II-2 evidence								
Rayner et al. (2012)[15]	Step-wise OL	*N* = 40 HD (71 including PD and CKDND) Kt/V: NR Males 77.5 % Median age 64 (35–88) Baseline: Ca 2.45 mmol/L PO4 1.54 mmol/L iPTH 25.3 pmol/L	Refractory to emollients or antihistamines 58 % of HD patients tried antihistamines	NR	Gabapentin 100 mg daily post-HD, adjusted to efficacy and tolerability; final median 700 mg/week Min: 100 mg post-HD Max: 900 mg daily	Significant reduction of itch 31 (77.5 %) Median follow-up: 2.5 months	5^b^	Unknown adverse event (8)
Chiekh Hassan et al. (2015)[16]	R, cohort	*N* = 13 HD with pruritus (15 total) Kt/V: NR Males 73.3 % Mean age 70.1 ± 10.6 Baseline: Ca 2.26 ± 0.2 mmol/L PO4 1.6 ± 0.5 mmol/L iPTH 16.8 pmol/L (IQR 11.3–23.9)	NR	Identifiable non-CKD cause of RLS or pruritus, incomplete/missing data, on gabapentin prior to attending institution	Gabapentin 100 mg q2d, adjusted by 25 mg for efficacy and tolerability Final average 90 mg/day	POS-S Renal score (pruritus): Baseline 17.9 Subsequent visits: NR, *p* < 0.05 Follow-up: 27 weeks	6^b^	Unsteadiness (2) Blurred vision (1)

*Ca* calcium, *CKD* chronic kidney disease, *CKDND* chronic kidney disease non-dialysis, *DB* double-blind, *HD* hemodialysis, *Hgb* hemoglobin, *h* hour(s), *iPTH* intact parathyroid hormone, *IQR* interquartile range, *Kt/V* number used to quantify hemodialysis treatment adequacy, *NR* not reported, *OL* open-label, *PC* placebo-controlled, *PD* peritoneal dialysis, *PO*_*4*_ phosphate, *R* randomized, *RCT* randomized controlled trial, *SB* single-blinded, *VAS* visual analog scale

^a^Jadad score (0–5)

^b^Newcastle Ottawa score (0–9)

Four studies reported no conflicts of interest [15–18] and the remaining three studies did not provide a statement of conflicts of interest [14, 19, 20]. Marquez et al. declared that gabapentin was supplied without charge from a local pharmaceutical company [14].

In general, the studies were small in size, ranging from 13 to 40 HD patients, and study designs were variable. Mean age ranged from 54 to 70 years and the percentage of males was variable. Where reported, HD was conducted three times weekly in all studies with the exception of the study by Naini et al. where patients are dialyzed twice weekly [19]. The mean Kt/V was only reported in four studies ranging from 1.23 to 1.37. Follow-up ranged from 4 to 27 weeks and was unreported in the study by Tol et al. [20]. Quality assessment of each study is reported in Table 1; overall, the initial agreement amongst the two assessors determined by kappa statistic was poor amongst the clinical studies (kappa = 0.167; poor agreement) and the kappa statistic for the cohort studies was perfect (kappa = 1.00; perfect agreement). After discussions, we determined the final reported quality scores as reported in Table 1.

Gabapentin dosing ranged from 100 mg post-HD (300 mg weekly) to 900 mg daily (6300 mg weekly). Five studies evaluated efficacy based on a visual analog scale (VAS) ranging from 0 to 10 with the exception of Razeghi et al. where a scale ranging from 0 to 100 was used [18]. The presence or absence of adverse events were reported in all studies and, when reported, were typically consistent with symptoms of central nervous system depression.

## Literature review

### Level I evidence

Gunal et al. studied 25 adult patients at a single center in Turkey on HD thrice weekly using a polysulfone dialyzer [17]. Patients did not have concomitant dermatological, liver, or metabolic diseases. Patients had refractory pruritus unresolved for greater than 8 weeks and had failed trials of antihistamines, nicergoline, or moisturizers. All antipruritic medications were discontinued for 1 week prior to treatment. Patients were randomized to receive 4 weeks of gabapentin 300 mg thrice weekly post-HD or placebo for 4 weeks in a double-blinded, cross-over fashion with a 1-week washout between treatments. Severity of pruritus was recorded on a VAS (0–10) once daily and the median score at each treatment period was used as the outcome score. A reduction in score of greater than or equal to 50 % was considered clinically significant.

All 25 patients completed the study with no dropouts. The mean Kt/V was 1.37 ± 0.35, and baseline mineral bone disease blood work were all within treatment target ranges based on the KDIGO 2009 recommendations [21]. From a baseline mean VAS of 8.4 ± 0.94, placebo decreased the mean score to 7.6 ± 2.6 (*p* = 0.098) with 4 patients achieving clinically significant improvement. Gabapentin decreased the mean score to 1.2 ± 1.8 (*p* = 0.001) with 1 patient failing to achieve a clinically significant response. The 1-week washout produced near-baseline VAS score of 7.9 ± 1.1. Although incidence was not reported, mild to moderate somnolence, dizziness, and fatigue that typically subsided within 7 days were commonly experienced with gabapentin.

Naini et al. randomized 34 patients from Iran on HD twice weekly with refractory pruritus to antihistamines and persistent symptoms for greater than 8 weeks [19]. Patients received either gabapentin 400 mg or placebo twice weekly post-HD for 4 weeks. All had normal liver enzyme tests and were reviewed by a dermatologist to ensure no evidence of other skin diseases. Antipruritic medications were discontinued 1 week prior to the study. Efficacy was assessed with a VAS (0–10) measured at the commencement of study and after each HD session. The mean decrease in pruritus scores after the study period was compared between the two groups.

Patient-specific baseline characteristics were not provided. After a baseline VAS score of 7.2 ± 2.3, a mean decrease of 6.7 ± 2.6 and 1.5 ± 1.8 in the gabapentin and placebo groups was found, respectively (*p* < 0.001). Separation of VAS scores between gabapentin and placebo were notable by the second HD session. The full antipruritic effects of gabapentin were approximately achieved by the sixth HD session. The most commonly reported side effects were mild to moderate somnolence, dizziness, and nausea, usually subsiding within 5–10 days from the first gabapentin dose. Incidence of side effects was not reported and no dropouts due to adverse events occurred.

Tol et al. studied 14 patients in a randomized, blinded, placebo-controlled cross-over trial at a single center in Turkey on HD three times weekly using a polysulfone dialyzer [20]. The type of blinding or how patients were randomized was not specified. The authors were contacted but we did not receive a reply by the time of publication. Patients were refractory to antihistamines, nicergoline, and moisturizers, with symptoms lasting for more than 8 weeks. Patients had no concomitant disease states associated with pruritus, and all antipruritic medications were discontinued 1 week before the study. The authors state that patients were assigned to receive 8 weeks of gabapentin 300 mg post-HD, followed by a placebo phase of unspecified duration, with a 1-week washout between the treatment phases. Patients recorded the severity of their pruritus on a VAS daily. Reduction in the VAS score by 50 % was considered clinically significant. Median pruritus VAS score at baseline and each treatment periods were measured. The study further evaluated sleep quality, depression, and quality of life using the modified post-sleep inventory, the Beck Depression Inventory, and the Medical Outcomes Study (SF-36), respectively.

All 14 patients completed the study and no adverse events were observed. Baseline calcium, phosphate, and PTH were within normal limits. Gabapentin decreased pruritus VAS score from a mean 7.6 ± 1.2 to 1.3 ± 1.4 (*p* = 0.01), post-sleep inventory decreased from 5.8 ± 3.3 to 1.8 ± 1.8 (*p* = 0.002), and Beck Depression Inventory decreased from 13.6 ± 5.2 to 7.1 ± 3.7 (*p* < 0.01). Both cognitive and somatic components of the Beck Depression Inventory were significantly reduced. Quality of life, as measured by both physical and mental components of the Medical Outcomes Study, improved from 45.1 ± 20.6 to 75.3 ± 11.4 and 56.9 ± 18.8 to 80.8 ± 10.3, respectively. Scores after the 1-week washout or placebo were not reported. No side effects were observed in this study.

### Level II-1 evidence

Razeghi et al. conducted a multicenter, double-blind, placebo-controlled, single-arm cross-over trial at 3 HD sites in Tehran, Iran [18]. Thirty-four patients undergoing HD thrice weekly, refractory to treatments with antihistamines and moisturizers, with ongoing pruritus for at least 2 weeks were treated. Gabapentin 100 mg post-HD was started for 4 weeks, then placebo for 4 weeks, with a 1-week washout in between the treatment periods. All patients were free from hepatic abnormalities with no skin lesions, metabolic diseases, drug allergies, or other disease states causing pruritus. Antipruritic drugs were stopped 1 week prior to the study. Patients were trained to use a VAS (0–100) daily to evaluate efficacy and the median score in each interval was used. A reduction in the score of 50 % or more was considered clinically significant.

After enrolment, 9 patients were excluded from the study: 2 for discontinuation due to adverse events (fatigue, dizziness, and drowsiness), 1 for inefficacy within 10 days, and 6 for poor compliance. Of the remaining 25 patients, 24 patients were found to exhibit distal symmetric polyneuropathy. Overall, none of the baseline parameters exhibited statistically significant change throughout the treatment phases of the study and were not significantly correlated with responsiveness of treatment including serum albumin, C- reactive protein (CRP), Kt/V, and phosphate levels. All patients had a baseline VAS of 100, decreasing to a mean score of 6.44 ± 8.46 (*p* < 0.001) with gabapentin and rising to 81.88 ± 11.06 during the placebo period (*p* < 0.001). Withdrawal of gabapentin during the 1-week washout period produced a mean VAS of 15 ± 11.27 (*p* < 0.001). Initial antipruritic effects were reported by approximately days 2–3, with maximal effects achieved by approximately days 24–25.

Marquez et al. investigated the efficacy and safety of gabapentin versus desloratadine at a single center in 19 adult patients undergoing HD thrice weekly using low-flux Polysulfone membranes in Argentina [14]. Potential patients were screened with a pruritus assessment tool on two occasions, 60 days apart. Those with persistent pruritus, defined as pruritus of any intensity occurring three times a week, were eligible for the study. All antipruritic agents were discontinued 1 week before the study. In a quasi-randomized open-label cross-over design, patients were assigned to either desloratadine 5 mg or gabapentin 300 mg three times weekly based on the dialysis schedule of each patient. Concomitant emollients or antipruritic agents were not permitted. Patients crossed over to the other treatment arm after 3 weeks with a 1-week washout in between treatment periods. The pruritus assessment tool, which included the pruritus VAS (0–10), was done at baseline and at the end of each treatment and washout periods. The questionnaire administrator was blinded to the drug assignment.

Ninety-two patients were screened: 22 were assigned to treatment groups and 19 completed the study. Baseline Kt/V, calcium, phosphate, and intact parathyroid hormone (iPTH) were within normal limits. From a baseline VAS for pruritus of 5.95 (range 4–8), gabapentin reduced the VAS to 4.6 (*p* = 0.07) compared to 3.44 (*p* = 0.004) with desloratadine. Although desloratadine had a greater reduction than gabapentin, the differences between the two agents when comparing the final VAS for each group were not statistically significant (*p* = 0.16). Desloratadine was more successful in producing clinically significant reductions (VAS reduction of at least 50 %) in 11 (58 %) patients compared with 5 (16 %) patients in the gabapentin group (*p* = 0.049). Nine (47 %) patients experienced fatigue and somnolence from gabapentin, leading to 4 discontinuations after the first dose. One subject on desloratadine withdrew because of nervousness.

### Level II-2 evidence

Rayner et al. studied 71 CKD non-dialysis (CKDND), peritoneal dialysis (PD), or HD patients in a single center in East Birmingham, England [15]. It was an open-label, uncontrolled trial to evaluate gabapentin and pregabalin for UP. No dialysis parameters were provided. Patients were enrolled in the study if the pruritus was troublesome, persistent, and refractory to emollients or antihistamines during routine consultations. Gabapentin was started at 100 mg post-HD and the dose was titrated according to symptoms by the patients in collaboration with their physician (ranging from 100 mg post-HD to 900 mg daily). Patients experiencing persistent pruritus or adverse events with gabapentin were offered pregabalin starting at 25 mg after HD.

A total of 40 consecutive HD patients were enrolled in the study with a median itching duration of 6 months; 88 % had sleep disturbances and 65 % had bleeding secondary to scratching. Baseline calcium, phosphate, and PTH were within normal limits. Baseline median severity of itch was 9 out of 10 measured with an unspecified tool. A median weekly dose of gabapentin 700 mg was reached at the end of the study with 31 (77.5 %) patients reporting a significant reduction in itch. Thirteen (32.5 %) patients’ final dose was 100 mg after dialysis, and 11 (27.5 %) received 100 mg daily. The remaining 16 (40 %) patients had final doses ranging from 300 mg post-HD to 900 mg daily. The authors reported use of a scale from 0 to 10 for measuring efficacy but did not provide the exact scale nor the specific median decrease in score in the HD group. The onset of action for gabapentin was reportedly after the first 1–2 doses or after up-titrations in dose. Eight patients discontinued gabapentin due to adverse events, and 1 patient discontinued due to inefficacy. Side effects specific to HD group were not reported, but the most common symptoms were over-sedation and dizziness. Four patients in the entire study who discontinued either gabapentin or pregabalin remained free of itch. Notably, 6 patients in the entire study achieved relief in concomitant pain conditions.

Cheikh Hassan et al. investigated the efficacy of gabapentin for UP and restless leg syndrome (RLS) in CKDND patients and sought to compare the results with HD patients at a single center in Sydney [16]. Efficacy of gabapentin was evaluated based on the validated Palliative care Outcome Scale-Symptoms (POS-S) Renal, which contains a pruritus score rated from 0 to 4. Dialysis parameters were unavailable. Gabapentin was started at 100 mg every 2 days in ESRD patients (CKD stages II–V). The authors did not define their classification of CKD stages II–V; we contacted the corresponding author for clarification but received no answer up until the time of publication. A pharmacist compounded 25-mg tablets for incremental titration.

Fifty-nine HD patients were referred to the center during the study period and 15 were enrolled (13 had symptoms of pruritus). Baseline calcium, phosphate, and PTH were within normal limits. The mean initial, daily average, and final gabapentin daily doses were 42.8, 90.0, and 128.6 mg respectively in HD patients. Although specific pruritus scores were not provided, the authors state that gabapentin was effective in reliving pruritus in HD patients between the first and subsequent visits to the clinic (*p* < 0.05). Two HD patients experienced adverse events (blurred vision and unsteadiness) compared with 16 patients in the CKDND group experiencing at least one adverse event. Univariate analyses were done in the CKDND group and none emerged to be associated with side effects including starting dose, average daily dose, and final dose.

## Discussion

Current evidence supports a trial of gabapentin for the relief of UP in HD patients refractory to antihistamines and/or moisturizers. Since the first systematic review by Vila et al., there have been five new studies published but none were randomized controlled trials. Six of seven studies in our systematic review reported favorable outcomes on pruritus from baseline after administering gabapentin orally at various oral doses ranging from 100 to 400 mg post-HD or 900 mg daily. One study found statistically significant benefits on sleep, depression, and quality of life [20] and another commented on improvements with pain [15]. Razeghi et al. had 24 of 25 patients experiencing neuropathy but did not comment on whether the improvement in pruritus was accompanied by improvement in neuropathy [18]. Only one study found no difference between gabapentin and baseline [14]. Overall, we support recommendations that gabapentin is a suitable alternative for generalized UP after failing antihistamines and/or topical emollients [6].

Adverse events including dizziness, fatigue, and somnolence were frequently reported across the studies, including one study where 9 (47 %) patients reported fatigue and somnolence, and 4 (21 %) discontinued gabapentin due to adverse events [14]. Renal clearance of gabapentin may vary in different dialysis patients depending on residual renal function and a lower starting dose is recommended for patients who are anuric. From the adverse events that were reported, they often subsided over 5–10 days [17, 19]. Although no serious neurotoxicities were found, their occurrences in this population have been reported in the literature, emphasizing the importance of renal dosing adjustments to mitigate this preventable risk [22, 23].

Although 6 positive gabapentin trials for UP suggest the drug is efficacious, it is important to discuss the various limitations, confounders, and biases in the included trials. Improving dialysis efficacy, frequency, and duration has not been firmly linked with improved UP outcomes, but the possibility must be considered [2]. Patients in the Marquez et al. study underwent dialysis using reused low-flux dialyzers, a practice that contrasts to the high-flux dialyzers used in North America [14]. HD was only scheduled twice weekly in the trial by Naini et al., and the Kt/V, which is recommended to be greater than 1.2, was reported to be an average of 1.23 ± 0.3 in the study by Marquez et al., raising the possibility of underdialysis in a proportion of the study population [14, 19]. Multiple studies did not report information about dialysis duration, frequency, and other parameters important in assessing adequacy [15, 16, 19].

Further, the included level II-1 and II-2 studies were generally of lower quality, making the interpretation of the subjective outcome of pruritus difficult. Four studies had a cross-over design with 1-week washout for each and thus the possibility of residual gabapentin effects encroaching the placebo phases cannot be excluded [14, 17, 18, 20]. Tol et al. state that patients were randomized to receive gabapentin, but no obvious concurrent comparator group or arm was stated [20]. Our inability to interpret the study design precludes our ability to fully evaluate the outcomes presented in the study. In Table 1, we have elected to describe study design as reported by the authors. The quasi-randomization in the study by Marquez et al. and the lower Kt/V of 1.23 ± 0.3 compared to other studies may have accounted for the lack of statistically significant improvement [14]. The patients in Marquez et al. also had a lower baseline pruritic VAS score of 5.95 compared to the other studies, and exposures were measured only once at the end of each treatment period and not multiple times within that period [14]. There may thus be a smaller role for gabapentin for patients with mild pruritus, but direct comparisons cannot be inferred. Various studies used mean VAS scores instead of medians, which may have skewed the data given the small study sizes. None of the studies explicitly stated whether the data was analyzed by intention-to-treat or per-protocol. Given the presence of dropouts in the studies by Rayner et al. and Marquez et al., analyzing the data by intention-to-treat would be the conservative approach [14, 15]. When assessing for bias, it was noted that carryover effects were seen in Razeghi et al. where post gabapentin washout, the pruritus score was significantly lower than prior to intervention. All cross-over studies used a 1-week washout between phases and though the medication is noted to have a half-life of 5 to 7 h in healthy individuals, there was a study (*n* = 11) that found an apparent elimination half-life of 132 h in anuric hemodialysis patients (24). This leads one to question whether 1 week is sufficient to ensure that there is no active medication in the serum. Finally, the method of administration of the various scales and tools were not explicitly stated across the studies. As severity of pruritus is subjective, variations in administration and the presence of investigators may significantly bias results.

## Conclusions

Our systematic review of seven studies supports the consideration of gabapentin for UP in HD patients refractory to first-line antihistamines and/or topical emollients, but the results should be interpreted cautiously due to the inclusion of generally lower quality studies. Alongside improvements in pruritus, gabapentin may additionally have a role in sleep, pain, quality of life, and depression in HD patients [15, 20]. If antihistamines and/or topical emollients are ineffective, we recommend considering starting gabapentin at the lowest possible dose of 100 mg orally post-HD and titrating slowly to minimize adverse events. Residual kidney function may contribute to variations in effective and tolerable dosages; Rayner et al. demonstrated that some patients may require dosages up to 900 mg daily [15]. Symptom improvement should be seen by 3 weeks and likely sooner [19]. The expected benefit is in the range of an absolute decrease of 5.7 to 9.4 points from baseline on a 10-point VAS scale by 3 to 8 weeks of medication use. Studies with titrations demonstrated that some patients may need higher dosages and would recommend titrations weekly depending on efficacy, tolerability, and availability of dosage forms. Side effects are often mild to moderate with this dosage and commonly occur within the first dose of medication and subsiding after 7 days.

Larger, well-designed, double-blinded, and controlled studies are required to minimize performance bias and placebo effect. Future studies may add to the literature by studying the additive efficacy of emollients and moisturizers to minimize the dosage of gabapentin or by comparing gabapentin with other agents like topical corticosteroids or more common antihistamines like diphenhydramine.

## Appendix

Sample search strategy for MEDLINE

1. exp Renal Dialysis/or exp renal replacement therapy/
2. (hemodialysis or haemodialysis).mp.
3. (hemodia* or haemodia* or hemofilt* or haemofilt* or diafilt*).mp.
4. dialysis.mp.
5. exp Hemofiltration/
6. or/1-5
7. exp Renal Insufficiency, Chronic/
8. exp Kidney Failure, Chronic/
9. (CKD or CRD or CRI or ESRD).mp.
10. kidney disease.mp.
11. renal disease.mp.
12. renal failure.mp.
13. kidney failure.mp.
14. 7 or 8 or 9 or 10 or 11 or 12 or 13
15. 6 or 14
16. exp Pruritus/
17. itch*.mp.
18. 16 or 17
19. exp gamma-Aminobutyric Acid/or (gabapentin or Neurontin or Fanatrex or FusePaq or Gralise).mp.
20. 15 and 18 and 19
